# Ageing in Asia: Beyond the Astana Declaration Towards Financing Long-term Care for All Comment on "Financing Long-term Care: Lessons From Japan"

**DOI:** 10.34172/ijhpm.2020.15

**Published:** 2020-02-18

**Authors:** Kai Hong Phua, Lee Gan Goh, Dina Sharipova

**Affiliations:** ^1^Graduate School of Public Policy, Nazarbayev Unversity, Nur-Sultan, Kazakhstan.; ^2^Lee Kuan Yew School of Public Policy, National University of Singapore, Singapore, Singapore.; ^3^Yong Loo Lin School of Medicine, National University of Singapore, Singapore, Singapore.

**Keywords:** Population Ageing, Long-term Care, Healthcare Financing, Astana Declaration, WHO Declaration on Primary Healthcare

## Abstract

The Astana Declaration on primary healthcare in 2018 was the attempt to revive the ideals of the World Health Organization (WHO) Alma-Ata Declaration 40 years later, together with a call for the political will to provide adequate financing at acceptable quality of care. This approach is taken to achieve the past ideals of Health for All, given the new challenges of universal health coverage. The economic case for primary healthcare is justified against the growing demand due in part to the growing costs of chronic conditions and the rise of ageing population, other than the supply-side factors of the healthcare industry. Past healthcare systems have evolved greater roles of the state versus the market, but few have involved the Third Sector or civil society in more integrated ways to provide and finance long-term care (LTC) with population ageing. From the extremes of the communist state to capitalist free markets, an optimal public-private system has to reach a balance in access, cost and quality for health and LTC. Recent studies of health and LTC have distilled newer developments in public-private mixes of provision, financing and regulation, in response to the needs of fast-ageing Asian societies. While Japan was the oldest country in the world, other countries in Asia have caught up and are now acknowledged where innovative models of integrated eldercare under economic limits, hold great promise of their transferability to the rest of ageing societies. Besides other forms of integrated LTC delivery with traditional systems, newer forms of financing like savings funds and superannuation have been developed, with participation from government, industry and civil society. There is much to learn from the new Asian models of financing, using appropriate technology and social innovations, and integrating health and social systems for LTC.

## Introduction

Forty years after the World Health Organization (WHO) Alma-Ata Declaration of primary healthcare, universal health for all has still to be achieved. The recent Astana Declaration in 2018 was updated to bring the ideals of the Alma-Ata Declaration to a realistic and strategic level to also include political will to commit adequate financing and to reach an acceptable quality of primary healthcare for all. Similarly, there is no adequate coverage for the ageing population without primary healthcare as enunciated in the Astana Declaration, without adequate financing and acceptable quality of primary healthcare for all countries, now and in the future.^1-3^ Recent analyses of population ageing have projected that the rising chronic needs are not sustainable with the existing health and long-term care (LTC) systems in developed and aged countries like Japan.^4^

## Role of Traditional Systems

Other than Japan, the newly industrialised economies that have developed social health insurance schemes like Taiwan, South Korea and Thailand, including those with a national health services in Hong Kong and Malaysia, are all coping with rising needs of the ageing population in chronic care and have called for increasing shift towards community and preventive care. But in addition to following the usual pathways of developing collective methods of providing health services by the state through taxation or insurance, these societies also had traditional forms that emphasized filial piety (Confucian societies in China, Korea, and Japan) and community participation in health-related activities (roles of clan associations, mutual funds and self-help projects based on the “kampung” village spirit of “gotong royong” in Southeast Asia or “bayanihan” in the Philippines). These fast- developing societies are evolving into new forms to balance the stresses of the modern world economy while preserving social structures, including eldercare by the community. Traditional systems of providing health and LTC can provide many lessons for modern systems as societies transition into advanced forms with changing technology.

However, countries in rapid transition are not just looking at the failed communist state or the excesses of the capitalist markets that have displaced these community organizations. They are still caught in struggles between the need to address post-colonial or post-communist legacies as well as attain socialist ideals to provide, finance and regulate health or welfare programmes. Besides tackling issues of poverty, inefficiencies, corruption or bureaucracies, they also rush to catch up with their more developed counterparts in the West. Given that these welfare state models are Pay-As-You-Go models whether tax-financed or insurance-based, as opposed to the expected market failures in predominantly private health insurance plans, the newly developed states in Asia are experimenting with newer alternative options that optimises between the state and market. Singapore, despite having strong government institutions, chose to operate within a mix of public and private tools that utilise the traditional forms of civil society such as family, community organizations and non-state, non-profit approaches to universal coverage and financing for a major societal issue such as ageing.

## The Third Sector

Nobel laureate Elinor Ostrom’s research such as in her book “*Governing the Commons,*” showed how common property can be managed successfully by the people who use them rather by governments or private companies, which challenged conventional wisdom that common resources can be successfully managed without government regulation or privatization.^5^ But can ageing issues and eldercare fall under the category of common pool resources in Ostrom’s Law and be subject to the same design principles for institutional economics? Can some of her design principles be applied to self-organized governance systems, including internal trust and reciprocity, and the nature of the resource system for ageing societies? In his seminal work, “Making Democracy Work,” Robert Putnam emphasized the importance of civil society organizations and community networks in promoting better government accountability and democratic performance.^6^ Later studies showed that non-governmental and community organizations can bring various positive social and economic outcomes. They facilitate cooperation and coordination for mutual benefits across different individuals as well as between the state and society.

Traditional community resources can contribute to better health and happiness of the population of various ages and fill the gap in case of state and market failures. In post-communist countries, after the collapse of the state and the break-down of command economy, the authorities had to significantly cut social benefits to their citizens due to dire economic crisis. As a result, people had to rely largely on informal help from their family members, friends, and communities rather than on the state. The reliance on community organizations is particularly critical for countries operating in weak institutional environments. Very often informal institutions, including immediate and extended family members, community and non-governmental organizations fill the gap left by the state. The emergence of the market economy in the 1990s with growing inequality and limited access to resources has not solved welfare problems, including that of older populations. In contrast, the capitalist market has left significant groups of society unequal in terms of their access to resources. In this case, community networks, families, and non-governmental organizations can therefore step in and be an important alternative to the state and capitalist market to cope with social problems, including those of the elderly.

A growing interest on the intersection between post-socialist developments and social policy emanates from complex issues pertaining to privatization of social care in Asia. Despite the introduction of universal health insurance, approaches of each country into the healthcare system significantly varies. In Asia, countries are concerned over social and economic growth that also led to related healthcare challenges amidst population changes in fertility and migration, besides ageing. With the rapid economic growth has come wealth and lifestyle changes but in their wake are emerging health issues due to increasing longevity. What are the implications for comparative health systems and policy research from the latest trends and issues in ageing Asia and beyond?^7,8^

Recent studies were able to document contemporary trends and policy issues to provide comparisons of social care systems undergoing rapid transitions, and to offer some examples of current best practices and lessons to meet changing needs due to population ageing in Asia.^9^ Fast-ageing, dynamic industrialising and urbanizing economies in Asia were selected for this regional comparative study – Japan, Korea, China, Hong Kong, Taiwan, and Singapore. Together, they presented a kaleidoscope of experiences learnt in coping with rapidly rising numbers of ageing persons in their midst. These can offer possible solutions to younger nations within and outside Asia, as they transition into the future to become aged and super-aged societies.^10^ In every country, there are found innovative ways and means to use public, private and voluntary sources to provide socially acceptable forms of traditional and modern care and have more sustainable forms of paying for these.

## Long-term Care

While the definition of LTC is debatable, it generally entails a range of services that aim to support elderly with functional and cognitive disability, either in the community or in residential facilities like nursing homes. There is a growing demand for LTC consequent to population ageing. As there is poor alignment of the financing framework between LTC and acute medical care, this generates perverse incentives for hospitalization. LTC services by private for-profit providers are underutilized, and support for informal caregivers are underdeveloped. Current government policies on LTC need to be revisited periodically, with a special focus on service delivery and financing. The affordability of LTC is attempted mainly through financing frameworks such as a combination of taxation and subsidies, insurance in covering catastrophic illness and disability, or mixes of these with innovative financing based on medical savings.^11^

Whilst the prevention, health promotion, and treatment strategies of primary healthcare are useful, they are not enough. LTC services are also needed for those elderly individuals who need assistance or institutional care. The social insurance models to pool risks for more sophisticated care in Germany, Japan and Korea provide some options and the experience was that such health financing systems should be set up early to avoid a system of ad-hoc decisions and inefficient expenditure later.^4^ Given the wide variety of possibilities and socio-cultural preferences, the different options for LTC in Asia are still being debated and evaluated on achieving an optimum balance of the roles of the public, private and people sectors to provide more sustainable models of social care. While newer transitional societies in post-socialist economies or developing societies with younger populations have not undergone demographic transitions in the rest of the world, these are projected to have ageing and aged societies in the future, by all accounts. Many of these newly emerging industrialised societies in developing Asia are studying the full impact within the Little Dragon or Tiger Economies and the emerging ASEAN countries, including strategies for economic development changes while putting into place social policies such as ageing and healthcare.^10,11^

## Long-term Care Financing

Much of the concerns over the limits of financing the ageing population looms from issues of sustaining huge outlays of welfare spending from the public budgets while private insurance is unaffordable. The traditional forms of public financing will have to be overhauled in terms of seeking alternatives between public and private sources, including tapping non-profit besides non-market options. However, this negates the optimization of public-private mixes in financing with regulatory balances between supply and demand while instituting more substitutes for medical care through social means in LTC. Funding sources from taxation and LTC insurance have been used normally but with more rationing, sustainability issues are concerned over the limits of financing.

How LTC services are delivered differ in each country. Whilst governments have tended to focus on the poor, Germany opted to make LTC universally available in 1995/1996. Japan followed this model but with one difference namely, but unlike the German model of those eligible opting for cash benefits, the Japanese model set up in 2000 is limited to services. The lessons learnt from the Japanese experience is “to introduce public LTC insurance at an early stage before benefits have expanded as a result of ad hoc policy decisions.”^4^ Elsewhere in Asia, new health insurance plans are introduced, from instituting personal savings plans to targeted co-payment in catastrophic insurance with public-private contributions, and with endowment rather than pay-as-you-go budgets, such as the mixed but integrated healthcare and LTC financing systems in Singapore are designed for the ageing society of the future.^9^

## Technology and Social Innovations

Many regional trends in technology and social innovation are developing in Asian countries to act as laboratories for innovations in ageing and where several drivers such as overuse of technology and lack of co-ordination across systems, could be addressed through suitable tools, policies, regulations and incentives. These include health technology assessment tools for cost control; co-ordination of care for older populations, supportive information systems, and financing mechanisms to encourage integration of services and technology. Increasingly these health technology assessment tools and physician payment methods are used not only in countries with national health insurances, but also in systems where private payments require guidelines or benchmarks from government authorities.

In terms of social innovations, three major trends were noted in pensions and financial security before they get “old,” they can benefit from the lessons learnt from the mistakes and successes of pension systems in older wealthy countries, by innovative pension system reforms to harmonise traditional cultural norms such as filial piety with the current realities of ageing in Asia. The use of integrated community health systems to reduce fragmentation of care by using allied health workers like social workers, care managers, as well as nurses, all working together as a multidisciplinary team. Regulation and policy are necessary constructs in the management of appropriate technology and social innovations. Regulation ensures public safety and at the same time, can direct local industries to develop new products and services. Expeditious regulatory reviews would encourage innovations and industry, while maintaining the production of cost-effective and affordable products. Finally, the point is made of overcoming ageism as the “largest social innovation” to enable all other innovations and policies to fall into place, and realizing a better world for all older people.^9^

## Discussion

While many aspects of the ageing phenomenon may be universal, there are nonetheless, highly specific and contextualized experiences for all the individual countries throughout Asia. In the ageing person, common problems that pertain to frailty and declining physical and mental functions are usually medicalized, as non-communicable diseases, vaccine preventable infections, and chronic problems all demand attention. Policies and programmes to change social behaviours for factors like smoking, sedentary lifestyle, obesity, hypertension, and diabetes, are critical in managing age-related conditions. A contextualized and holistic life course approach of ageing forms the personal agenda of action as each society has unique experiences to share. While Japan was the oldest country in the world, other countries in Asia have caught up and are now acknowledged where innovative models of integrated urban living for ageing populations under economic limits hold great promise of their transferability to the rest of ageing Asia.

## Conclusions

The vison of Health for All through primary healthcare, expressed in the Declaration of Alma Ata in 1978, is yet to be achieved for the whole world. Countries in Asia have made progress with primary healthcare towards Health for All. Countries like Japan and China also applied primary care principles of healthy lifestyle in the elderly and benefitted through better health status in their aged populations. The Declaration of Astana in 2018 affirmed the commitment to continue the vision of health for all through primary care, universal health coverage, and the United Nations 2030 Agenda for Sustainable Development in pursuit of Health for All. Fulfilling the Declaration of Astana to prevent disease and promote health in the elderly will not be enough. There is a need for LTC services for those who have disabilities arising out of ageing processes. There is much to learn from the new Asian models of financing, using appropriate technology and social innovations and integrating health and LTC systems.

## Ethical issues

Not applicable.

## Competing interests

Authors declare that they have no competing interests.

## Authors’ contributions

KHP is the principal author who conceptualized the commentary, covering the policy framework for a “whole of society” approach to the balanced provision and financing of LTC for population ageing, while LGG wrote on the integration of medical and social care systems in Asia for holistic LTC, and DS gave social science perspectives for a trans-disciplinary commentary.

## Authors’ affiliations

^1^Graduate School of Public Policy, Nazarbayev Unversity, Nur-Sultan, Kazakhstan. ^2^Lee Kuan Yew School of Public Policy, National University of Singapore, Singapore, Singapore. ^3^Yong Loo Lin School of Medicine, National University of Singapore, Singapore, Singapore.
